# Self-reported help-seeking behavior in the event of mental disorders among adults in Poland

**DOI:** 10.3389/fpubh.2025.1612066

**Published:** 2025-06-12

**Authors:** Aleksandra Lewandowska, Mateusz Jankowski, Mariusz Gujski, Paulina Mularczyk-Tomczewska, Agata Szulc, Andrzej Silczuk

**Affiliations:** ^1^Children Psychiatry Unit Specialized Psychiatric Health Care Centre in Lodz, Lodz, Poland; ^2^Department of Population Health, Centre of Postgraduate Medical Education, Warsaw, Poland; ^3^Department of Public Health, Medical University of Warsaw, Warsaw, Poland; ^4^Psychiatric Clinic, Faculty of Health Sciences, Medical University of Warsaw, Warsaw, Poland; ^5^Department of Environmental Psychiatry, Faculty of Life Sciences, Medical University of Warsaw, Warsaw, Poland

**Keywords:** mental health, mental disorders, psychiatry, health services, mental health literacy

## Abstract

**Introduction:**

The decision to use psychological or psychiatric help is complex and depends on many interrelated factors. This study aimed to characterize self-reported help-seeking behavior in the event of mental disorders among adults in Poland.

**Methods:**

This questionnaire-based cross-sectional survey was carried out in March 2025 using computer-assisted web interviews (CAWI) among 1,114 adults in Poland. Quota-sampling method was used.

**Results:**

Most of the respondents (54.8%) declared that they would be able to recognize symptoms related to mental disorders on their own, and 50.7% declared that they would be able to recognize symptoms related to mental disorders in others and recognize people who require the help of a psychiatrist or psychologist. Over two-thirds of respondents (67.1%) declared that they know where to seek help if they experience mental disorders on their own and 61.4% declared that they know where to seek help if a child or teenager is experiencing mental disorders. Only 17.2% of respondents declared that enough activities are carried out in Poland to promote mental health and prevent mental disorders. In multivariable logistic regression, female gender, ever visit to a specialist due to mental disorders and a history of mental disorders in the close family were the most important factors associated (*p* < 0.05) with self-reported help-seeking behavior in the event of mental disorders.

**Conclusion:**

This study revealed significant gaps in self-reported help-seeking behavior in the event of mental disorders among adults in Poland. Public health interventions are needed to strengthen mental health literacy among adults in Poland, particularly concerning help-seeking behavior.

## 1 Introduction

Mental disorders are serious global public health problems, affecting the functioning of millions of people worldwide (1). The presence of mental disorders significantly reduces the quality of life of patients – both physical, social, and emotional (1, 2). About 7.3% of the global burden of disease is attributed to mental and behavioral disorders (2). Most of this burden is related to unipolar depressive disorders, as well as anxiety, psychotic, and substance use disorders (2). Currently, about 450 million people suffer from such disorders and it is predicted that one in four people worldwide will be affected by a mental disorder at some point in their life (2). People with mental disorders experience disproportionately higher rates of disability and mortality (3). People with major depressive disorder and schizophrenia had a 40 to 60% higher risk of premature death than the general population (4). Studies show that most people struggling with mental disorders encounter numerous barriers in accessing healthcare (5, 6). The process of seeking help is often associated with fear of rejection, self-stigma, and negative perception by society (7, 8). As a result, many people with mental disorders do not receive support on time, which leads to delayed diagnosis and treatment, deterioration of health, as well as an increased risk of social exclusion, and additional costs – both personal and systemic (9). In recent years, a growing number of publications on mental disorders has been observed in the public domain. However, many people struggling with emotional difficulties, anxiety, depression, or other mental disorders still decide not to contact a specialist (8). Often, months or even years pass before a person experiencing mental suffering reaches out for professional support (9). Individual barriers in recognizing the need for treatment and in reaching out for specialist support are strongly related to the level of knowledge, beliefs, and attitudes toward mental health and available forms of help. Mental health symptoms, such as irritability, sleep problems, sadness, or fatigue, can be misinterpreted as physical problems, making it difficult to properly recognize the problem. As a result, patients may not adopt adequate coping strategies or delay the decision to seek help (10). People who have more knowledge about their illness and are convinced of the benefits of treatment are more willing to seek support (9, 11). Researchers indicate that intersectional characteristics, such as place of residence, gender, or socioeconomic status, can significantly influence help-seeking behavior. For example, women from rural areas may encounter limitations in accessing services, while men, especially in cultures promoting self-sufficiency, may avoid seeking help for fear of losing their position or autonomy (12, 13). Additionally, factors such as poverty, lack of time, overload with duties, or communication limitations can significantly hinder access to treatment (13–16). Despite these barriers, a literature review indicates that patients’ health aspirations—the desire to improve their quality of life and well-being—often exceed the difficulties in accessing medical care (9, 10, 17).

People with mental disorders may be exposed to stigmatization, that can lead to a sense of isolation, fear of being judged, and even experiences of discrimination (7, 18). Such social attitudes significantly reduce patients’ motivation to seek professional help (19). At the family and local community level, the involvement of loved ones in the treatment process plays an important role. Available research results indicate that active support from the family and educational activities aimed at local communities can facilitate open conversations about mental health and increase patients’ willingness to use available forms of help. Patients and their families were less motivated to seek help when the healthcare system perceived them as complicated and difficult to access, and its mode of operation was not consistent with their beliefs and level of understanding (20). The decision to use psychological or psychiatric help is complex and depends on many interrelated factors. Awareness of one’s problems, lack of stigma, availability of services, social support, as well as individual psychological characteristics and cultural beliefs—all these elements can affect willingness to seek help (20). Understanding these mechanisms is crucial not only for mental health practitioners but also for health policymakers, teachers, parents, and all those who have a real impact on shaping a supportive and open social environment. Actions aimed at strengthening mental health literacy at the European level may contribute to the rising public awareness of mental disorders (21, 22).

It is estimated that in 2020, 91% of countries in the WHO European region had mental healthcare plans and policies (MHPPs) (23). Eastern Europe is indicated as a high-priority European region where post-graduate training, regional collaboration, and capacity building on mental disorders should be implemented (24). Literature on “mental health literacy” is growing rapidly but mainly in high-income countries (25). Adolescents and young adults are often listed as priority populations for mental health literacy programs and campaigns (26, 27).

Structural barriers such as service availability and waiting lists may also influence public attitudes toward help-seeking behaviors (28–30). The low number of psychiatrists is listed as a major barrier to mental health services and relatively long wait times in Poland (29, 30). To address gaps in access to mental health services, the National Mental Health Protection Programme provides access to new forms of mental health services, including Mental Health Centres, that guarantee access to mental services on time (29). Data on self-reported help-seeking behavior may provide practical implications for the ongoing reform and improvement of the psychiatric care system in Poland.

This study aimed to characterize self-reported help-seeking behavior in the event of mental disorders among adults in Poland.

## 2 Materials and methods

### 2.1 Study design

A nationwide cross-sectional survey was conducted over the period of March 8–10, 2025, using the computer-assisted web interviewing (CAWI) methodology. The instrument utilized in the study was a standardized questionnaire developed by the research team to capture relevant information aligned with the study objectives. Data acquisition was outsourced to an experienced public opinion polling agency (Ariadna) (31), which facilitated participant recruitment and managed the digital infrastructure for questionnaire deployment. Respondents were invited to participate via individual email messages containing a unique survey link, with subsequent SMS reminders sent to maximize response rates.

Participants were recruited from a large, pre-verified online panel comprising more than 100,000 individuals. Sampling followed a stratified quota-based approach, with stratification parameters including sex, age, and urbanization level of place of residence. These parameters were based on current national demographic distributions as reported by Statistics Poland (32). The overall response rate was estimated at 22%. To maintain sampling integrity, if a respondent declined participation, another panel member matching the same stratification profile was invited in their place. Respondents were obligated to answer all the questions, so there were no missing answers.

This sampling strategy is consistent with that employed in prior population-level health and behavioral studies conducted in Poland (33, 34), allowing for methodological comparability across different survey waves. The sample size was calculated based on the demographic structure of the adult population of Poland and the number of participants was calculated as at least 1,000 individuals.

The study protocol was approved by the Ethics Committee of the Medical University of Warsaw (decision AKBE/38/2025 as of 24 Feb 2025) and the study followed the Helsinki Declaration rules. Participation in this study was voluntary and anonymous, informed consents were collected. All procedures followed the regulations expressed in the Declaration of Helsinki.

### 2.2 Description of the participant sample

A total of 1,114 adults took part in this survey, 54.7% were females. Among the participants, 13.6% were aged 18–29 years, 19.5% 30–39 years, 19.1% 40–49 years, 19.5% 50–59 years and 28.4% were over 60. Less than half of respondents (45.6%) had higher education and 38% lived in rural areas. Among the participants, 26.1% had children under 18 years old, 15.7% lived alone, 36.4% lived with one person, and 47.8% lived with at least two people. Most of the respondents were currently employed or self-employed (active occupational status; 61.5%) and 47% declared a good household financial situation.

### 2.3 Measures

Self-reported help-seeking behavior in the event of mental disorders was assessed using four different questions. The questionnaire was self-prepared based on a literature review (12, 13, 18, 35). An expert group (including national consultants for psychiatry) reviewed the questionnaire. The pilot study was conducted, and 8 adult individuals filled out questionnaires twice, 5 days apart. After this pilot study, two questions were revised to better address the study objective and reduce the risk of bias.

Respondents were asked about their self-reported ability to recognize symptoms of mental disorders using the following questions: (1) “In your opinion, would you be able to recognize symptoms related to mental disorders in yourself?” and (2) “In your opinion, would you be able to recognize symptoms related to mental problems in others and recognize people who require the help of a psychiatrist or psychologist?,” each question with 5-point Likert scale.

Respondents were also asked about their ability to seek help when experiencing mental disorders: (1) “Do you know where to seek help if you experience mental disorders?” and (2) “Do you know where to seek help if a child or teenager is experiencing mental disorders?,” each question with 5-point Likert scale.

In the 4 abovementioned questions, responses “definitely yes” and “rather yes” were combined into one variable, to analyze sociodemographic differences and identify factors associated with higher competencies related to self-reported help-seeking behaviors.

Moreover, one question on educational campaigns on mental disorders was addressed: “Do you think that enough activities are carried out in Poland to promote mental health and prevent mental disorders?,” with a 5-point Likert scale. In this question, responses “definitely no” and “rather no” were combined into one variable, to analyze sociodemographic differences and identify groups that perceive an insufficient number of educational campaigns on mental disorders.

### 2.4 Statistical analysis

All statistical analyses were performed using IBM SPSS Statistics version 29 (Armonk, NY, United States). Descriptive statistics included frequencies and percentages to present the distribution of categorical variables. Comparisons between categorical variables were conducted using contingency tables and chi-square tests. Associations between 10 different variables and help-seeking behavior in the event of mental disorders among adults in Poland were analyzed using the multivariable logistic regression analyses. Each variable was first assessed independently using bivariable logistic regression. Variables that demonstrated statistical significance in these bivariable analyses were subsequently entered into multivariable logistic regression models. Associations were expressed as odds ratios (OR) with corresponding 95% confidence intervals (95% CI). A *p*-value of less than 0.05 was considered indicative of statistical significance.

## 3 Results

### 3.1 Self-reported help-seeking behaviors in the event of mental disorders

Most of the respondents declared that they would be able (46% rather yes and 8.8% definitely yes) to recognize symptoms related to mental disorders on their own (Table 1). Moreover, half of the respondents (50.7%) declared that they would be able (43.2% rather yes and 7.5% definitely yes) to recognize symptoms related to mental disorders in others and recognize people who require the help of a psychiatrist or psychologist. Over two-thirds of respondents (67.1%) declared that they know where to seek help if they experience mental disorders on their own (Table 1). Among the respondents, 61.4% declared that they know where to seek help if a child or teenager is experiencing mental disorders. Only 17.2% of respondents (14.5% rather yes and 2.7% definitely yes) declared that enough activities are carried out in Poland to promote mental health and prevent mental disorders (Table 1). Moreover, among the respondents 23.2% had ever visited psychiatrists, 26.8% had ever visited psychologists and 15.9% had ever visited psychotherapists (Table 1). History of mental disorders diagnosed by a doctor in a close family (parents, siblings, children, spouse) was declared by 21.1% of respondents (Table 1).

**Table 1:** Self-reported help-seeking behavior in the event of mental disorders among adults in Poland (*n* = 1,114).

Variable	n	%
In your opinion, would you be able to recognize symptoms related to mental disorders in yourself?		
Definitely yes	98	8.8
Rather yes	512	46.0
Rather no	204	18.3
Definitely no	56	5.0
I do not know	244	21.9
In your opinion, would you be able to recognize symptoms related to mental problems in others and recognize people who require professional help?		
Definitely yes	83	7.5
Rather yes	481	43.2
Rather no	194	17.4
Definitely no	58	5.2
I do not know	298	26.8
Do you know where to seek help if you experience mental disorders?		
Definitely yes	171	15.4
Rather yes	576	51.7
Rather no	172	15.4
Definitely no	49	4.4
I do not know	146	13.1
Do you know where to seek help if a child or teenager is experiencing mental disorders?		
Definitely yes	144	12.9
Rather yes	540	48.5
Rather no	169	15.2
Definitely no	69	6.2
I do not know	192	17.2
Do you think that enough activities are carried out in Poland to promote mental health and prevent mental disorders?		
Definitely yes	30	2.7
Rather yes	162	14.5
Rather no	425	38.2
Definitely no	279	25.0
I do not know	218	19.6
Have you ever sought help from a specialist due to mental disorders?		
Yes, psychiatrist	259	23.2
Yes, psychologist	298	26.8
Yes, psychotherapist	177	15.9
History of mental disorders diagnosed by a doctor in close family		
Yes	235	21.1
No	879	78.9

### 3.2 Sociodemographic differences in self-reported help-seeking behavior in the event of mental disorders

There were statistically significant differences in self-reported help-seeking behavior in the event of mental disorders by sociodemographic variables (Tables 2–6).

**Table 2:** Factors associated with ability to recognize symptoms related to mental disorders in yourself (*n* = 1,114).

In your opinion, would you be able to recognize symptoms related to mental disorders in yourself? – “rather yes” or “definitely yes”						
			Bivariable logistic regression		Multivariable logistic regression	
Variable	%	*p*	OR (95%CI)	p	aOR (95%CI)	*p*
Gender						
Female (*n* = 609)	60.1	**<0.001**	1.61 (1.27–2.05)	**<0.001**	1.51 (1.18–1.93)	**0.001**
Male (*n* = 505)	48.3		Reference		Reference	
Age [years]						
18–29 (*n* = 151)	58.9	0.6	1.28 (0.87–1.90)	0.2		
30–39 (*n* = 217)	51.6		0.95 (0.67–1.35)	0.8		
40–49 (*n* = 213)	55.4		1.11 (0.78–1.57)	0.6		
50–59 (*n* = 217)	57.1		1.19 (0.84–1.69)	0.3		
60 + (*n* = 316)	52.8		Reference			
Educational level						
Higher education	57.5	0.1	1.22 (0.97–1.55)	0.1		
Less than higher	52.5		Reference			
Place of residence						
Rural area (*n* = 423)	49.2	**0.02**	Reference		Reference	
City below 20,000 residents (*n* = 143)	54.5		1.24 (0.85–1.81)	0.3	1.10 (0.74–1.63)	0.6
City from 20,000 to 99,999 residents (*n* = 218)	62.4		1.71 (1.23–2.39)	**0.002**	1.59 (1.13–2.24)	**0.01**
City from 100,000 to 499,999 residents (*n* = 187)	55.1		1.27 (0.90–1.79)	0.2	1.19 (0.83–1.70)	0.3
City ≥ 500,000 residents (*n* = 143)	59.4		1.52 (1.03–2.23)	0.03	1.36 (0.91–2.02)	0.1
Having children under 18 years old						
Yes (*n* = 291)	53.3	0.6	0.92 (0.71–1.21)	0.6		
No (*n* = 823)	55.3		Reference			
Number of household members						
Living alone (*n* = 175)	57.1	0.8	1.12 (0.79–1.58)	0.5		
2 (*n* = 406)	54.2		0.99 (0.77–1.28)	0.9		
3 or more (*n* = 533)	54.4		Reference			
Professional status						
Active (*n* = 685)	54.7	0.9	0.99 (0.78–1.27)	0.9		
Passive (*n* = 429)	54.8		Reference			
Self-reported economic status						
Good (*n* = 524)	59.9	**<0.001**	1.15 (0.80–1.66)	0.5		
Moderate (*n* = 436)	47.9		0.71 (0.49–1.03)	0.07		
Bad (*n* = 154)	56.5		Reference			
Ever visit to specialist (psychiatrist, psychologist, or psychotherapist) due to mental disorders						
Yes (*n* = 388)	68.3	**<0.001**	2.38 (1.84–3.08)	**<0.001**	2.01 (1.54–2.63)	**<0.001**
No (*n* = 726)	47.5		Reference		Reference	
History of mental disorders in close family						
Yes (*n* = 235)	71.5	**<0.001**	2.48 (1.81–3.39)	**<0.001**	1.97 (1.42–2.73)	**<0.001**
No (*n* = 879)	50.3		Reference		Reference	

OR, odds ratio; aOR, adjusted odds ration; 95%CI, 95-percent confidence interval. Statisitcally significant values are bolded.

**Table 3:** Factors associated with ability to recognize symptoms related to mental disorders in others and recognize people who require professional help (*n* = 1,114).

In your opinion, would you be able to recognize symptoms related to mental problems in others and recognize people who require professional help? – “rather yes” or “definitely yes”						
			Bivariable logistic regression		Multivariable logistic regression	
Variable	%	*p*	OR (95%CI)	*p*	aOR (95%CI)	*p*
Gender						
Female (*n* = 609)	55.3	**<0.001**	1.52 (1.20–1.92)	**<0.001**	1.39 (1.08–1.78)	**0.01**
Male (*n* = 505)	45.0		Reference		Reference	
Age [years]						
18–29 (*n* = 151)	59.6	**0.03**	1.86 (1.25–2.75)	**0.002**	1.47 (0.97–2.22)	0.07
30–39 (*n* = 217)	50.7		1.29 (0.91–1.83)	0.1	1.13 (0.79–1.62)	0.5
40–49 (*n* = 213)	53.1		1.42 (1.01–2.01)	**0.04**	1.30 (0.90–1.87)	0.2
50–59 (*n* = 217)	51.2		1.32 (0.93–1.86)	0.1	1.17 (0.82–1.68)	0.4
60 + (*n* = 316)	44.3		Reference		Reference	
Educational level						
Higher education	54.5	**0.02**	1.33 (1.05–1.69)	**0.02**	1.31 (1.02–1.68)	**0.04**
Less than higher	47.4		Reference		Reference	
Place of residence						
Rural area (*n* = 423)	48.2	0.6	Reference			
City below 20,000 residents (*n* = 143)	48.3		1.00 (0.69–1.46)	0.9		
City from 20,000 to 99,999 residents (*n* = 218)	54.1		1.27 (0.91–1.76)	0.2		
City from 100,000 to 499,999 residents (*n* = 187)	51.3		1.13 (0.80–1.60)	0.5		
City ≥ 500,000 residents (*n* = 143)	53.8		1.25 (0.86–1.83)	0.2		
Having children under 18 years old						
Yes (*n* = 291)	53.6	0.2	1.18 (0.90–1.54)	0.2		
No (*n* = 823)	49.6		Reference			
Number of household members						
Living alone (*n* = 175)	48.0	0.5	0.83 (0.59–1.17)	0.3		
2 (*n* = 406)	49.3		0.88 (0.68–1.14)	0.3		
3 or more (*n* = 533)	52.5		Reference			
Professional status						
Active (*n* = 685)	51.8	0.3	1.13 (0.89–1.44)	0.3		
Passive (*n* = 429)	48.7		Reference			
Self-reported economic status						
Good (*n* = 524)	56.1	**<0.001**	1.21 (0.85–1.74)	0.3		
Moderate (*n* = 436)	43.8		0.74 (0.51–1.07)	0.1		
Bad (*n* = 154)	51.3		Reference			
Ever visit to specialist (psychiatrist, psychologist, or psychotherapist) due to mental disorders						
Yes (*n* = 388)	64.9	**<0.001**	2.46 (1.91–3.17)	**<0.001**	2.05 (1.57–2.68)	**<0.001**
No (*n* = 726)	43.0		Reference		Reference	
History of mental disorders in close family						
Yes (*n* = 235)	67.7	**<0.001**	2.45 (1.81–3.32)	**<0.001**	1.95 (1.42–2.68)	**<0.001**
No (*n* = 879)	46.1		Reference		Reference	

OR, odds ratio; aOR, adjusted odds ration; 95%CI, 95-percent confidence interval. Statisitcally significant values are bolded.

**Table 4:** Factors associated with knowledge where to seek help if experience own mental disorders (*n* = 1,114).

Do you know where to seek help if you experience mental disorders? – “rather yes” or “definitely yes”						
			Bivariable logistic regression		Multivariable logistic regression	
Variable	%	*p*	OR (95%CI)	*p*	aOR (95%CI)	*p*
Gender						
Female (*n* = 609)	73.1	**<0.001**	1.82 (1.42–2.35)	**<0.001**	1.71 (1.32–2.22)	**<0.001**
Male (*n* = 505)	59.8		Reference		Reference	
Age [years]						
18–29 (*n* = 151)	65.6	0.05	Reference			
30–39 (*n* = 217)	63.1		0.90 (0.58–1.39)	0.6		
40–49 (*n* = 213)	62.4		0.87 (0.57–1.35)	0.5		
50–59 (*n* = 217)	67.3		1.08 (0.70–1.68)	0.7		
60 + (*n* = 316)	73.4		1.45 (0.96–2.20)	0.08		
Educational level						
Higher education	69.7	0.09	1.25 (0.97–1.60)	0.09		
Less than higher	64.9		Reference			
Place of residence						
Rural area (*n* = 423)	64.3	0.4	0.83 (0.55–1.24)	0.4		
City below 20,000 residents (*n* = 143)	64.3		0.83 (0.51–1.35)	0.5		
City from 20,000 to 99,999 residents (*n* = 218)	70.6		1.11 (0.70–1.75)	0.7		
City from 100,000 to 499,999 residents (*n* = 187)	70.1		1.07 (0.67–1.72)	0.8		
City ≥ 500,000 residents (*n* = 143)	68.5		Reference			
Having children under 18 years old						
Yes (*n* = 291)	65.3	0.5	0.90 (0.68–1.19)	0.5		
No (*n* = 823)	67.7		Reference			
Number of household members						
Living alone (*n* = 175)	65.7	0.3	1.02 (0.71–1.46)	0.9		
2 (*n* = 406)	70.0		1.24 (0.94–1.63)	0.1		
3 or more (*n* = 533)	65.3		Reference			
Professional status						
Active (*n* = 685)	66.0	0.3	0.88 (0.68–1.14)	0.3		
Passive (*n* = 429)	68.8		Reference			
Self-reported economic status						
Good (*n* = 524)	71.4	**0.01**	1.27 (0.87–1.87)	0.2		
Moderate (*n* = 436)	62.2		0.84 (0.57–1.23)	0.4		
Bad (*n* = 154)	66.2		Reference			
Ever visit to specialist (psychiatrist, psychologist, or psychotherapist) due to mental disorders						
Yes (*n* = 388)	79.1	**<0.001**	2.46 (1.85–3.28)	**<0.001**	2.09 (1.55–2.80)	**<0.001**
No (*n* = 726)	60.6		Reference		Reference	
History of mental disorders in close family						
Yes (*n* = 235)	82.6	**<0.001**	2.79 (1.94–4.01)	**<0.001**	2.23 (1.53–3.25)	**<0.001**
No (*n* = 879)	62.9		Reference		Reference	

OR, odds ratio; aOR, adjusted odds ration; 95%CI, 95-percent confidence interval. Statisitcally significant values are bolded.

**Table 5:** Factors associated with knowledge where to seek help if a child or teenager is experiencing mental disorders (*n* = 1,114).

Do you know where to seek help if a child or teenager is experiencing mental disorders? – “rather yes” or “definitely yes”						
			Bivariable logistic regression		Multivariable logistic regression	
Variable	%	*p*	OR (95%CI)	*p*	aOR (95%CI)	*p*
Gender						
Female (*n* = 609)	66.8	**<0.001**	1.66 (1.30–2.12)	**<0.001**	1.59 (1.24–2.03)	**<0.001**
Male (*n* = 505)	54.9		Reference		Reference	
Age [years]						
18–29 (*n* = 151)	64.9	0.7	1.13 (0.76–1.70)	0.5		
30–39 (*n* = 217)	59.0		0.88 (0.62–1.25)	0.5		
40–49 (*n* = 213)	58.7		0.87 (0.61–1.24)	0.4		
50–59 (*n* = 217)	63.1		1.05 (0.73–1.50)	0.8		
60 + (*n* = 316)	62.0		Reference			
Educational level						
Higher education	63.4	0.2	1.17 (0.92–1.49)	0.2		
Less than higher	59.7		Reference			
Place of residence						
Rural area (*n* = 423)	60.3	0.06	1.01 (0.68–1.48)	0.9		
City below 20,000 residents (*n* = 143)	52.4		0.73 (0.50–1.17)	0.2		
City from 20,000 to 99,999 residents (*n* = 218)	65.1		1.24 (0.80–1.91)	0.3		
City from 100,000 to 499,999 residents (*n* = 187)	67.4		1.37 (0.87–2.15)	0.2		
City ≥ 500,000 residents (*n* = 143)	60.1		Reference			
Having children under 18 years old						
Yes (*n* = 291)	67.0	**0.02**	1.39 (1.05–1.84)	**0.02**	1.39 (1.04–1.85)	**0.03**
No (*n* = 823)	59.4		Reference		Reference	
Number of household members						
Living alone (*n* = 175)	61.7	0.5	0.95 (0.67–1.34)	0.8		
2 (*n* = 406)	59.1		0.85 (0.65–1.11)	0.2		
3 or more (*n* = 533)	63.0		Reference			
Professional status						
Active (*n* = 685)	61.2	0.8	0.98 (0.76–1.25)	0.8		
Passive (*n* = 429)	61.8		Reference			
Self-reported economic status						
Good (*n* = 524)	64.3	0.2	1.18 (0.82–1.71)	0.4		
Moderate (*n* = 436)	58.3		0.92 (0.63–1.33)	0.6		
Bad (*n* = 154)	60.4		Reference			
Ever visit to specialist (psychiatrist, psychologist, or psychotherapist) due to mental disorders						
Yes (*n* = 388)	70.1	**<0.001**	1.79 (1.38–2.32)	**<0.001**	1.52 (1.16–1.99)	**0.003**
No (*n* = 726)	56.7		Reference		Reference	
History of mental disorders in close family						
Yes (*n* = 235)	76.6	**<0.001**	2.44 (1.75–3.39)	**<0.001**	2.08 (1.48–2.92)	**<0.001**
No (*n* = 879)	57.3		Reference		Reference	

OR, odds ratio; aOR, adjusted odds ration; 95%CI, 95-percent confidence interval. Statisitcally significant values are bolded.

**Table 6:** Factors associated with opinion that there are not enough activities are carried out in Poland to promote mental health and prevent mental (*n* = 1,114).

Do you think that enough activities are carried out in Poland to promote mental health and prevent mental disorders? – “rather no” or “definitely no”						
			Bivariable logistic regression		Multivariable logistic regression	
Variable	%	*p*	OR (95%CI)	*p*	aOR (95%CI)	*p*
Gender						
Female (*n* = 609)	66.3	**0.02**	1.35 (1.06–1.72)	**0.02**	1.28 (0.99–1.64)	0.05
Male (*n* = 505)	59.4		Reference		Reference	
Age [years]						
18–29 (*n* = 151)	60.9	0.4	0.77 (0.51–1.14)	0.2		
30–39 (*n* = 217)	60.4		0.75 (0.52–1.07)	0.1		
40–49 (*n* = 213)	64.8		0.90 (0.63–1.30)	0.6		
50–59 (*n* = 217)	60.4		0.75 (0.52–1.07)	0.1		
60 + (*n* = 316)	67.1		Reference			
Educational level						
Higher education	70.6	**<0.001**	1.85 (1.44–2.38)	**<0.001**	1.81 (1.40–2.33)	**<0.001**
Less than higher	56.8		Reference		Reference	
Place of residence						
Rural area (*n* = 423)	58.9	0.06	Reference		Reference	
City below 20,000 residents (*n* = 143)	63.6		1.22 (0.83–1.81)	0.3	1.16 (0.77–1.72)	0.5
City from 20,000 to 99,999 residents (*n* = 218)	61.9		1.14 (0.81–1.59)	0.5	1.06 (0.75–1.49)	0.8
City from 100,000 to 499,999 residents (*n* = 187)	69.5		1.59 (1.11–2.30)	**0.01**	1.40 (0.96–2.04)	0.08
City ≥ 500,000 residents (*n* = 143)	69.2		1.57 (1.05–2.36)	**0.03**	1.31 (0.86–2.00)	0.2
Having children under 18 years old						
Yes (*n* = 291)	59.1	0.09	0.79 (0.60–1.04)	0.09		
No (*n* = 823)	64.6		Reference			
Number of household members						
Living alone (*n* = 175)	65.1	**0.01**	1.31 (0.92–1.87)	0.1	1.26 (0.87–1.82)	0.2
2 (*n* = 406)	68.2		1.51 (1.15–1.98)	**0.003**	1.55 (1.17–2.04)	**0.002**
3 or more (*n* = 533)	58.7		Reference		Reference	
Professional status						
Active (*n* = 685)	62.2	0.4	0.89 (0.70–1.15)	0.4		
Passive (*n* = 429)	64.8		Reference			
Self-reported economic status						
Good (*n* = 524)	66.6	0.08	1.38 (0.96–2.00)	0.09		
Moderate (*n* = 436)	60.6		1.06 (0.73–1.55)	0.8		
Bad (*n* = 154)	59.1		Reference			
Ever visit to specialist (psychiatrist, psychologist, or psychotherapist) due to mental disorders						
Yes (*n* = 388)	66.2	0.1	1.22 (0.95–1.59)	0.1		
No (*n* = 726)	61.6		Reference			
History of mental disorders in close family						
Yes (*n* = 235)	70.2	**0.01**	1.49 (1.09–2.03)	**0.01**	1.45 (1.05–1.99)	**0.02**
No (*n* = 879)	61.3		Reference		Reference	

OR, odds ratio; aOR, adjusted odds ration; 95%CI, 95-percent confidence interval. Statisitcally significant values are bolded.

In multivariable logistic regression, female gender (aOR: 1.51 [1.18–1.93]; *p* = 0.001), living in a city from 20,000 to 99,999 residents (aOR: 1.59 [1.13–2.24]; *p* = 0.01), ever visit to a specialist due to mental disorders (aOR: 2.01 [1.54–2.63]; *p* < 0.001] and history of mental disorders in the close family (aOR: 1.97 [1.42–2.73]; *p* < 0.001) were significantly associated with higher ability to recognize symptoms related to mental disorders in yourself (Table 2). Female gender (aOR: 1.39 [1.08–1.78]; *p* = 0.01), higher educational level (aOR: 1.31 [1.02–1.68]; *p* = 0.04), ever visit to a specialist due to mental disorders (aOR: 2.05 [1.57–2.68]; *p* < 0.001] and history of mental disorders in the close family (aOR: 1.95 [1.42–2.68]; *p* < 0.001) were significantly associated with higher ability to recognize symptoms related to mental disorders in others and recognize people who require the help of a psychiatrist or psychologist (Table 3). Female gender (aOR: 1.71 [1.32–2.22]; *p* < 0.001), ever visit to a specialist due to mental disorders (aOR: 2.09 [1.55–2.80]; *p* < 0.001] and history of mental disorders in the close family (aOR: 2.23 [1.53–3.25]; p < 0.001) were significantly associated with knowledge where to seek help if experience own mental disorders (Table 4). Female gender (aOR: 1.59 [1.24–2.03]; *p* < 0.001), having children under 18 years old (aOR: 1.39 [1.04–1.85]; *p* = 0.03), ever visit a specialist due to mental disorders (aOR: 1.52 [1.16–1.99]; *p* = 0.003] and history of mental disorders in the close family (aOR: 2.08 [1.48–2.92]; *p* < 0.001) were significantly associated with knowledge where to seek help if a child or teenager is experiencing mental disorders (Table 5). Having higher education (aOR: 1.81 [1.40–2.33]; p < 0.001), living with one household member (aOR: 1.55 [1.17–2.04]; *p* = 0.002) and history of mental disorders in the close family (aOR: 1.45 [1.05–1.99]; *p* = 0.02) were significantly associated with opinion that there are not enough activities are carried out in Poland to promote mental health and prevent mental (Table 6).

## 4 Discussion

This is the first nationwide study on self-reported help-seeking behavior in the event of mental disorders among adults in Poland. Findings from this study showed that almost half of adults in Poland are not able to recognize symptoms related to mental disorders, both in themselves and in others. Moreover, approximately one-third of adults in Poland did not know where to seek help by professional mental health care, both for adults or children and teenagers. This study also showed that 44.6% of adults in Poland declared an insufficient number of actions on mental health promotion and prevention of mental disorders. Female gender, ever visit to a specialist due to mental disorders and a history of mental disorders in the close family were the most important factors associated (*p* < 0.05) with self-reported help-seeking behavior in the event of mental disorders.

Seeking mental health services is often a complex and emotionally difficult process, and people with mental disorders may encounter numerous barriers in accessing healthcare (5–8). Providing easy access to mental health services and public education on the mental health care system are key actions to reduce the gap in the number of people with mental disorders who seek help. Lui et al. showed that self-reliance, stigma, and poor mental health literacy are key barriers to help-seeking for common mental disorders among university students (36). Good mental health literacy and social encouragement are significant facilitators to help-seeking for common mental disorders (36). Similar barriers and facilitators were reported in the study by Noorwali et al. (37). Public stigma and lack of awareness, unprofessional mental health practitioners, and lack of accessibility to services and information were listed as major barriers, wherein, e.g., societal and family awareness, promoting the accessibility of services, enhancing sources of external support were listed as facilitators to help-seeking for mental disorders (37). In the study by Elshaikh et al. carried out among older adults, stigma, negative beliefs about mental health professional services, and cost were the most reported barriers to professional mental health help-seeking (38). Prior positive experiences with mental health services and high socioeconomic status were the most important facilitators of help-seeking (38). Tunks et al. (39) reported that stigma and patients’ perceptions and understandings of common mental disorders impacted their help-seeking decision-making and engagement with service through primary care (39).

Females declared better ability to recognize symptoms related to mental disorders in themselves or others (especially relatives). Moreover, females more often This observation may result from the fact that females often present higher health literacy skills (40, 41), as well as from the sociocultural role of females in families and society. Respondents who lived in cities from 20,000 to 99,999 residents were more likely to declare their ability to recognize symptoms related to mental disorders in themselves. This observation may result from the fact that in small local urban communities, educational activities on mental health may have higher effectiveness. Respondents with higher education were more likely to declare their ability to recognize symptoms related to mental problems in others and recognize people who require professional help. This observation may result from the self-assessment and self-perception of people with higher education when compared to other people, especially those without higher education. As expected, those who had children were more likely to declare knowledge and seek help if a child or teenager was experiencing mental disorders. This observation may result from the fact that parents are gatekeepers to mental health services for children and those who have children are more up-to-date with current mental health services available for children in Poland (42).

In this study, there was no significant impact of socioeconomic status on self-reported help-seeking behavior in the event of mental disorders. This observation suggests that sociodemographic variables have a limited impact on help-seeking behavior in the event of mental disorders, as mental disorders can affect people from different socioeconomic groups. Moreover, there were no age differences in self-reported help-seeking behavior in the event of mental disorders, which may result from the fact that mental disorders pose a populational problem, and various age groups are exposed to risk factors for different mental disorders.

This is the first study on self-reported help-seeking behavior in the event of mental disorders among adults in Poland, so direct comparisons with national studies are impossible. However, findings from this study showed that ever visit to a specialist due to mental disorders and a history of mental disorders in the close family was significantly associated with self-reported help-seeking behavior in the event of mental disorders. Findings from this study are comparable to those reported by Noorwali et al., (37) and Elshaikh et al. (38), who also reported that prior positive experience with mental health services (38) and family awareness of mental disorders (37) are facilitators to help-seeking for mental disorders. Contrary to previously published data (37–39), in this study female gender was associated with self-reported help-seeking behavior in the event of mental disorders. However, this observation is in line with nationwide studies in Poland on public awareness of different health conditions, as numerous of those studies pointed out gender differences in health-related behaviors, with females as a group more likely to present higher health literacy levels (43–45).

In this study, one-third of participants did not know where to seek help in the event of mental health problems, both their own and those of minors. This lack of knowledge could be linked to poor mental health literacy or insufficient informational campaigns, which should be highlighted as a priority for public health interventions and further analyses.

In this study, respondents were also asked about their opinions on mental health promotion and prevention activities that were carried out in Poland. Mental health promotion and prevention programs may reduce the risk of stigma and encourage people to seek professional help during mental health crises (46, 47). In this study, 44.6% of respondents declared that there are insufficient health promotion and prevention actions in Poland. Moreover, the insufficient number of educational campaigns on mental health may affect trust in the health system and willingness to seek help behaviors. Media mental health awareness campaigns may play a significant role in strengthening mental health literacy levels (48, 49). Also, social media can be used as an effective communication channel for mental health campaigns (50). This observation underlines the need to increase the number of public health interventions for mental health promotion and prevention.

The following practical implications are resulting from this study. First, this study showed significant gaps in self-reported help-seeking behavior in the event of mental disorders among adults in Poland. Public health interventions are needed to strengthen mental health literacy among adults in Poland. Second, over one-third of adults in Poland are not aware of where to seek help when experiencing mental disorders, so media campaigns on the organization of mental health care in Poland are needed. The development of media campaigns, targeted to particular sociodemographic groups, may contribute to the improvement of mental health literacy levels. Third, general practitioners as those healthcare professionals who are closest to the patient, should be more involved in the education of patients on the mental health care system in Poland. The training of primary care professionals is key to strengthening the preparedness of healthcare workers for public health challenges resulting from the growing incidence of mental disorders. School and community programs focused on children and adolescents are essential to address mental health literacy gaps in younger generations. Longitudinal studies to evaluate how perceptions and help-seeking behaviors evolve over time are also needed.

### 4.1 Limitations

There are several limitations related to this study. Self-reported help-seeking behavior in the event of mental disorders was assessed based on 4 questions that were prepared by the authors based on the literature review. However, different types of questions can be addressed to assess help-seeking behaviors. There was no formal validation of the questionnaire. As there is no standardized questionnaire on help-seeking behavior in the event of mental disorders, we decided to use self-prepared questions. Second, the CAWI method was used so that only those with internet access could participate in this study. Third, as this is a cross-sectional survey, re-call bias may occur. Online recruitment may pose some risk of selection bias. Social desirability bias should also be mentioned. Moreover, the history of visits to mental health professionals and the history of mental disorders in the family were self-reported and medical documentation was not verified. The lack of detailed clinical information that can be compared with responses from the questionnaire is a limitation typical for cross-sectional surveys.

## 5 Conclusion

This study revealed significant gaps in self-reported help-seeking behavior in the event of mental disorders among adults in Poland. Almost half of adults in Poland were not able to recognize symptoms of mental disorders and one-third of adults were not aware of where to seek mental health care. Most of the sociodemographic factors did not influence help-seeking behavior, and female gender, ever visiting a specialist due to mental disorders and a history of mental disorders in the close family were the most important factors associated with self-reported help-seeking behavior in the event of mental disorders. Public health interventions including media campaigns, school-based interventions, and training programs for healthcare professionals are needed to develop health literacy competencies and address current gaps in self-reported help-seeking behavior.

## Data Availability

The raw data supporting the conclusions of this article will be made available by the authors, without undue reservation.

## References

[1] World Health Organization. WHO highlights urgent need to transform mental health and mental health care (2022). Available online at: https://www.who.int/news-room/releases/17-06-2022-who-highlights-urgent-need-to-transform-mental-health-and-mental-health-care (Accessed April 10, 2025).

[2] GBD 2019 Mental Disorders Collaborators. Global, regional, and national burden of 12 mental disorders in 204 countries and territories, 1990-2019: a systematic analysis for the global burden of disease study 2019. Lancet Psychiatry. (2022) 9:137–50. doi: 10.1016/S2215-0366(21)00395-3, PMID: 35026139 PMC8776563

[3] Ferrari AJ Somerville AJ Baxter AJ Norman R Patten SB Vos T. Global variation in the prevalence and incidence of major depressive disorder: a systematic review of the epidemiological literature. Psychol Med. (2013) 43:471–81. doi: 10.1017/S0033291712001511, PMID: 22831756

[4] Saxena S Funk M Chisholm D. World health assembly adopts comprehensive mental health action plan 2013-2020. Lancet. (2013) 381:1970–1. doi: 10.1016/S0140-6736(13)61139-3, PMID: 23746771

[5] Andrade LH Alonso J Mneimneh Z Wells JE al-Hamzawi A Borges G. Barriers to mental health treatment: results from the WHO world mental health surveys. Psychol Med. (2014) 44:1303–17. doi: 10.1017/S0033291713001943, PMID: 23931656 PMC4100460

[6] Yap MB Reavley N Jorm AF. Where would young people seek help for mental disorders and what stops them? Findings from an Australian national survey. J Affect Disord. (2013) 147:255–61. doi: 10.1016/j.jad.2012.11.014, PMID: 23228570

[7] Bee P Brooks H Fraser C Lovell K. Professional perspectives on service user and carer involvement in mental health care planning: a qualitative study. Int J Nurs Stud. (2015) 52:1834–45. doi: 10.1016/j.ijnurstu.2015.07.008, PMID: 26253574 PMC4642654

[8] Cruwys T Gunaseelan S. “depression is who I am”: mental illness identity, stigma and wellbeing. J Affect Disord. (2016) 189:36–42. doi: 10.1016/j.jad.2015.09.012 26402345

[9] Wang XQ Wang Y Yu K Ma R Zhang JY Zhou YQ. Experiences of care-seeking by schizophrenia patients with delayed diagnosis and treatment in rural China: a qualitative study. Int J Soc Psychiatry. (2023) 69:1453–61. doi: 10.1177/00207640231164010, PMID: 37036139

[10] Godoy L Hodgkinson S Robertson HA Sham E Druskin L Wambach CG. Increasing mental health engagement from primary care: the potential role of family navigation. Pediatrics. (2019) 143:e20182418. doi: 10.1542/peds.2018-2418, PMID: 30877145

[11] Hailemariam M Fekadu A Prince M Hanlon C. Engaging and staying engaged: a phenomenological study of barriers to equitable access to mental healthcare for people with severe mental disorders in a rural African setting. Int J Equity Health. (2017) 16:156. doi: 10.1186/s12939-017-0657-0, PMID: 28851421 PMC5576237

[12] Hailemariam M Fekadu A Selamu M Medhin G Prince M Hanlon C. Equitable access to integrated primary mental healthcare for people with severe mental disorders in Ethiopia: a formative study. Int J Equity Health. (2016) 15:121. doi: 10.1186/s12939-016-0410-0, PMID: 27460038 PMC4962424

[13] Isaacs AN Maybery D Gruis H. Help seeking by aboriginal men who are mentally unwell: a pilot study. Early Interv Psychiatry. (2013) 7:407–13. doi: 10.1111/eip.12015, PMID: 23347361

[14] Abayneh S Lempp H Alem A Alemayehu D Eshetu T Lund C. Service user involvement in mental health system strengthening in a rural African setting: qualitative study. BMC Psychiatry. (2017) 17:187. doi: 10.1186/s12888-017-1352-9, PMID: 28521749 PMC5437561

[15] Mall S Hailemariam M Selamu M Fekadu A Lund C Patel V. Restoring the person’s life’: a qualitative study to inform development of care for people with severe mental disorders in rural Ethiopia. Epidemiol Psychiatr Sci. (2017) 26:43–52. doi: 10.1017/S2045796015001006, PMID: 26961343 PMC6998647

[16] Hancock N Smith-Merry J Mckenzie K. Facilitating people living with severe and persistent mental illness to transition from prison to community: a qualitative exploration of staff experiences. Int J Ment Health Syst. (2018) 12:45. doi: 10.1186/s13033-018-0225-z, PMID: 30116292 PMC6085690

[17] Muhorakeye O Biracyaza E. Exploring barriers to mental health services utilization at Kabutare District Hospital of Rwanda: perspectives from patients. Front Psychol. (2021) 12:638377. doi: 10.3389/fpsyg.2021.638377, PMID: 33828506 PMC8019821

[18] Reddy SK Thirthalli J Channaveerachari NK Reddy KN Ramareddy RN Rawat VS. Factors influencing access to psychiatric treatment in persons with schizophrenia: a qualitative study in a rural community. Indian J Psychiatry. (2014) 56:54–60. doi: 10.4103/0019-5545.124714, PMID: 24574559 PMC3927246

[19] Angdembe M Kohrt BA Jordans M Rimal D Luitel NP. Situational analysis to inform development of primary care and community-based mental health services for severe mental disorders in Nepal. Int J Ment Health Syst. (2017) 11:69. doi: 10.1186/s13033-017-0176-9, PMID: 29167700 PMC5688643

[20] Zhang L Chen Y Li Q Zhang J Zhou Y. Barriers and facilitators to medical help-seeking in rural patients with mental illness: a qualitative Meta-synthesis. Asian Nurs Res (Korean Soc Nurs Sci). (2024) 18:203–14. doi: 10.1016/j.anr.2024.04.010, PMID: 38704085

[21] World Health Organization. Strengthening mental health in the WHO European region in 2024: a year in review. Available online at:: https://www.who.int/europe/publications/i/item/WHO-EURO-2025-11229-51001-51001#:~:text=The%20year%202024%20saw%20the%20Mental%20Health%20Flagship%2C,areas%3A%20policy%2C%20services%2C%20data%20collection%20and%20stigma%20reduction (Accessed May 25, 2025).

[22] World Health Organization. Guidance on mental health policy and strategic action plans. Module 5. Comprehensive directory of policy areas, directives, strategies and actions for mental health. Geneva: World Health Organization; (2025) (Guidance on mental health policy and strategic action plans). Available online at: https://iris.who.int/bitstream/handle/10665/380469/9789240106871-eng.pdf?sequence=1 (Accessed May 25, 2025).

[23] Guerrero Z Kågström A Aliev A Tomášková H Yon Y Lazeri L. Mental health plans and policies across the WHO European region. Glob Ment Health. (2024) 11:e110. doi: 10.1017/gmh.2024.88, PMID: 39588205 PMC11588412

[24] Hook K Bogdanov S. Mental health care in Eastern Europe and Central Asia: an analysis of needs and a call for greater investment. Lancet Reg Health Eur. (2021) 10:100182. doi: 10.1016/j.lanepe.2021.100182, PMID: 34806062 PMC8589706

[25] Sweileh WM. Global research activity on mental health literacy. Middle East Curr Psychiatry. (2021) 28:43. doi: 10.1186/s43045-021-00125-5

[26] Sun G Wang C Zhang J. Effectiveness of mental health literacy interventions for adolescents: a systematic review and meta-analysis. SAGE Open. (2025) 15. doi: 10.1177/21582440251327445 [Epub ahead of print].

[27] Nobre J Oliveira AP Monteiro F Sequeira C Ferré-Grau C. Promotion of mental health literacy in adolescents: a scoping review. Int J Environ Res Public Health. (2021) 18:9500. doi: 10.3390/ijerph18189500 34574427 PMC8470967

[28] Gao Y Burns R Leach L Chilver MR Butterworth P. Examining the mental health services among people with mental disorders: a literature review. BMC Psychiatry. (2024) 24:568. doi: 10.1186/s12888-024-05965-z, PMID: 39164690 PMC11334396

[29] Sagan A Kowalska-Bobko I Biechowska D Rogala M Gałązka-Sobotka M. Implementation of mental health Centres pilots in Poland since 2018: a chance to move towards community-based mental health services. Int J Environ Res Public Health. (2022) 19:5774. doi: 10.3390/ijerph19095774, PMID: 35565173 PMC9099713

[30] Klingemann J Bieńkowski P Misiak B Samochowiec J. Mental healthcare and addiction services in Poland: a (hi)story of attraction and rejection. Int Rev Psychiatry. (2024):1–12. doi: 10.1080/09540261.2024.242781938557342

[31] Arianda Poland’s Research Panel. About the panel. Available online at: https://panelariadna.com/ (Accessed April 10, 2025).

[32] Statistics Poland. Demographic yearbook of Poland (2024). Available online at: https://stat.gov.pl/en/topics/statistical-yearbooks/statistical-yearbooks/demographic-yearbook-of-poland-2024,3,18.html (Accessed April 10, 2025).

[33] Ostrowska A Jankowski M Pinkas J. Public support for car smoking bans in Poland: a 2022 national cross-sectional survey. BMJ Open. (2022) 12:e066247. doi: 10.1136/bmjopen-2022-066247, PMID: 36216427 PMC9557322

[34] Kamińska A Pinkas J Jankowski M. Factors associated with the frequency of eye examinations among adults in Poland - a nationwide cross-sectional survey, December 2022. Ann Agric Environ Med. (2023) 30:287–95. doi: 10.26444/aaem/159152, PMID: 37387379

[35] Lewandowska A Silczuk A Mularczyk-Tomczewska P Duda-Zalewska A Jankowski M Gujski M. Awareness of mental disorders and their risk factors - a nationwide crosssectional survey among adults in Poland. Front Psych. (2025) 16:1599683. doi: 10.3389/fpsyt.2025.1599683 PMC12146334 40491679

[36] Lui JC Sagar-Ouriaghli I Brown JSL. Barriers and facilitators to help-seeking for common mental disorders among university students: a systematic review. J Am Coll Heal. (2024) 72:2605–13. doi: 10.1080/07448481.2022.2119859, PMID: 36084266

[37] Noorwali R Almotairy S Akhder R. Barriers and facilitators to mental health help-seeking among young adults in Saudi Arabia: a qualitative study. Int J Environ Res Public Health. (2022) 19:2848. doi: 10.3390/ijerph19052848, PMID: 35270539 PMC8909985

[38] Elshaikh U Sheik R Saeed RKM Chivese T Alsayed Hassan D. Barriers and facilitators of older adults for professional mental health help-seeking: a systematic review. BMC Geriatr. (2023) 23:516. doi: 10.1186/s12877-023-04229-x, PMID: 37626290 PMC10463345

[39] Tunks A Berry C Strauss C Nyikavaranda P Ford E. Patients’ perspectives of barriers and facilitators to accessing support through primary care for common mental disorders in England: a systematic review. J Affect Disord. (2023) 338:329–40. doi: 10.1016/j.jad.2023.06.035 37348656

[40] Blom S Lindh F Lundin A Burström B Hensing G Löve J. How gender and low mental health literacy are related to unmet need for mental healthcare: a cross-sectional population-based study in Sweden. Arch Public Health. (2024) 82:12. doi: 10.1186/s13690-023-01228-7, PMID: 38273389 PMC10809616

[41] Clouston SAP Manganello JA Richards M. A life course approach to health literacy: the role of gender, educational attainment and lifetime cognitive capability. Age Ageing. (2017) 46:493–9. doi: 10.1093/ageing/afw229, PMID: 27940567 PMC5405758

[42] Villatoro AP Dupont-Reyes MJ Phelan JC. Parental recognition of preadolescent mental disorders: does stigma matter? Soc Sci Med. (2018) 216:88–96. doi: 10.1016/j.socscimed.2018.09.040 30273777 PMC6383650

[43] Sękowski K Grudziąż-Sękowska J Pinkas J Jankowski M. Public knowledge and awareness of diabetes mellitus, its risk factors, complications, and prevention methods among adults in Poland-a 2022 nationwide cross-sectional survey. Front Public Health. (2022) 10:1029358. doi: 10.3389/fpubh.2022.1029358, PMID: 36620244 PMC9810624

[44] Jaroń K Grajek M Kobza J Rozmiarek M. Assessing health literacy among hospitalized patients: a cross-sectional study in Silesia, Poland. Healthcare (Basel). (2025) 13:593. doi: 10.3390/healthcare13060593, PMID: 40150444 PMC11941875

[45] Moczeniat G Jankowski M Duda-Zalewska A Gujski M. Awareness of genitourinary cancers risk factors-a 2024 population-based cross-sectional study in Poland. Int J Public Health. (2024) 69:1607264. doi: 10.3389/ijph.2024.1607264, PMID: 38974046 PMC11224143

[46] Singh V Kumar A Gupta S. Mental health prevention and promotion-a narrative review. Front Psych. (2022) 13:898009. doi: 10.3389/fpsyt.2022.898009, PMID: 35958637 PMC9360426

[47] Sharma A Sharma SD Sharma M. Mental health promotion: a narrative review of emerging trends. Curr Opin Psychiatry. (2017) 30:339–45. doi: 10.1097/YCO.0000000000000347, PMID: 28661906

[48] Tam MT Wu JM Zhang CC Pawliuk C Robillard JM. A systematic review of the impacts of media mental health awareness campaigns on young people. Health Promot Pract. (2024) 25:907–20. doi: 10.1177/15248399241232646, PMID: 38468568 PMC11370183

[49] Duthie G Reavley N Wright J Morgan A. The impact of media-based mental health campaigns on male help-seeking: a systematic review. Health Promot Int. (2024) 39:daae104. doi: 10.1093/heapro/daae104, PMID: 39224087 PMC11369358

[50] Draganidis A Fernando AN West ML Sharp G. Social media delivered mental health campaigns and public service announcements: a systematic literature review of public engagement and help-seeking behaviours. Soc Sci Med. (2024) 359:117231. doi: 10.1016/j.socscimed.2024.117231, PMID: 39278158

